# The hemodynamic response function as a type 2 diabetes biomarker: a data-driven approach

**DOI:** 10.3389/fninf.2023.1321178

**Published:** 2024-01-05

**Authors:** Pedro Guimarães, Pedro Serranho, João V. Duarte, Joana Crisóstomo, Carolina Moreno, Leonor Gomes, Rui Bernardes, Miguel Castelo-Branco

**Affiliations:** ^1^University of Coimbra, Coimbra Institute for Biomedical Imaging and Translational Research (CIBIT), Institute for Nuclear Sciences Applied to Health (ICNAS), Coimbra, Portugal; ^2^Department of Sciences and Technology, Universidade Aberta, Lisbon, Portugal; ^3^University of Coimbra, Faculty of Medicine (FMUC), Coimbra, Portugal; ^4^Department of Endocrinology, University Hospital of Coimbra (CHUC), Coimbra, Portugal; ^5^University of Coimbra, Clinical Academic Center of Coimbra (CACC), Faculty of Medicine (FMUC), Coimbra, Portugal

**Keywords:** type 2 diabetes, functional magnetic resonance imaging, neuroimaging, deep learning, hemodynamic response

## Abstract

**Introduction:**

There is a need to better understand the neurophysiological changes associated with early brain dysfunction in Type 2 diabetes mellitus (T2DM) before vascular or structural lesions. Our aim was to use a novel unbiased data-driven approach to detect and characterize hemodynamic response function (HRF) alterations in T2DM patients, focusing on their potential as biomarkers.

**Methods:**

We meshed task-based event-related (visual speed discrimination) functional magnetic resonance imaging with DL to show, from an unbiased perspective, that T2DM patients’ blood-oxygen-level dependent response is altered. Relevance analysis determined which brain regions were more important for discrimination. We combined explainability with deconvolution generalized linear model to provide a more accurate picture of the nature of the neural changes.

**Results:**

The proposed approach to discriminate T2DM patients achieved up to 95% accuracy. Higher performance was achieved at higher stimulus (speed) contrast, showing a direct relationship with stimulus properties, and in the hemispherically dominant left visual hemifield, demonstrating biological interpretability. Differences are explained by physiological asymmetries in cortical spatial processing (right hemisphere dominance) and larger neural signal-to-noise ratios related to stimulus contrast. Relevance analysis revealed the most important regions for discrimination, such as extrastriate visual cortex, parietal cortex, and insula. These are disease/task related, providing additional evidence for pathophysiological significance. Our data-driven design allowed us to compute the unbiased HRF without assumptions.

**Conclusion:**

We can accurately differentiate T2DM patients using a data-driven classification of the HRF. HRF differences hold promise as biomarkers and could contribute to a deeper understanding of neurophysiological changes associated with T2DM.

## 1 Introduction

Type 2 diabetes mellitus (T2DM) is a prevalent chronic metabolic disorder with an enormous global impact. In 2021, approximately 537 million adults aged 20–79 had diabetes, a number expected to reach 783 million by 2045 due to rising obesity and sedentary lifestyles (IDF Diabetes Atlas, 2021). It is a significant burden on healthcare systems, as disease management and complications can be costly.

T2DM is a systemic disease impacting multiple organs and tissues. In the brain, it is associated with an increased risk of function loss and long-term cognitive impairment (Biessels et al., 2014; Biessels and Despa, 2018). Patients are at increased risk for cerebrovascular disease, dementia, and depression, among other neuropathies (Biessels et al., 2014; Bancks et al., 2017; Biessels and Despa, 2018). To support research and preventive therapies development, it’s important to study the neurophysiological changes associated with early brain dysfunction in T2DM subjects before overt vascular or structural lesions.

Although there is no consensus on their full extent and mechanisms, T2DM-related changes in the brain have been documented (Biessels and Reijmer, 2014; Chen et al., 2015; Duarte et al., 2015; Crisóstomo et al., 2021; Zhang et al., 2022; Duarte et al., 2023), with most literature focused on structural differences. Among the observed changes, magnetic resonance imaging (MRI) shows both local and general abnormalities in T2DM subjects, with a wide prevalence of cortical and subcortical gray matter atrophy, as shown by volumetric analysis at the whole-brain level and voxel-based morphometry (Moran et al., 2013; Moheet et al., 2015). Cortical thickness was found to be reduced in T2DM patients compared to controls (Chen et al., 2015; Crisóstomo et al., 2021). Differences in cortical gyrification were also associated with T2DM, mainly in cortical sensory areas (Crisóstomo et al., 2021).

Functional brain changes have also been described. However, these reports are rather inconsistent. Both task-based and resting state studies of blood flow and oxygenation level have been used to uncover neuropathological mechanisms in T2DM. However, resting state studies are much more common. Cerebral blood flow was found to be altered in T2DM patients in Ryan et al. (2014), but with a larger sample size, no statistically significant differences were found when accounting for brain volume (Tiehuis et al., 2008).

Functional magnetic resonance imaging (fMRI) with blood oxygen level-dependent (BOLD) contrast can indirectly quantify neural activity. Widespread abnormal activity has been reported in T2DM (Chhatwal and Sperling, 2012; Xia et al., 2013; Cui et al., 2014; Liu et al., 2018, 2019; Xiong et al., 2020). However, the pathophysiological mechanisms leading to structural changes, behavioral impairment, and brain dysfunction are still not well understood. Deconvolution analysis was used to examine the fMRI response profile in Duarte et al. (2015, 2023). Differences were found between controls and lesion-free T2DM patients, in this approach which considered the hemodynamic response function of capillary networks. However, data driven approaches are needed to validate these findings.

Brain imaging has become ubiquitous in neuroscience and mostly with model-driven approaches. In parallel, deep learning (DL) has shown incredible potential in medical image analysis (Litjens et al., 2017). Unsurprisingly, the neuroimaging and DL fusion has been promptly adopted by research groups around the world (Litjens et al., 2017; Zaharchuk et al., 2018; Warren and Moustafa, 2023). fMRI-based research has seen a surge in neural network (NN) applications. In Sarraf et al. (2016), scans from controls and Alzheimer’s disease patients were concatenated into 2D images and classified using convolutional NNs (CNNs). In Horikawa and Kamitani (2017), CNNs were used to decode seen and imagined objects. DL has also been used with resting state fMRI to classify chronic pain conditions (Santana et al., 2019), mild cognitive impairment (Suk et al., 2016), or schizophrenia (Kim et al., 2016).

In this work, we use a combination of task-based fMRI and DL to investigate whether T2DM patients have an altered BOLD response compared to age- and gender-matched controls. In contrast to typical fMRI studies, we used an event-related design, as this is the only design suitable to study an altered hemodynamic response function (HRF). This is of particular importance in the case of diabetes, where the assumption of a standard HRF is not guaranteed. We explore the explainability of DL methods to improve decision transparency and combine novel DL-based methods and deconvolution generalized linear model (GLM) analysis to reveal the true HRF in T2DM subjects without any assumptions.

## 2 Materials and methods

### 2.1 Ethics statement

This study was approved by the Ethics Committee of the Faculty of Medicine of the University of Coimbra (ethical approval number CE-059-2019). All participants provided informed written consent. The Helsinki Declaration of 1975 (revised in 1983) guidelines were followed throughout.

### 2.2 Participants

Recruited T2DM patients were diagnosed at the Endocrinology Department of the University of Coimbra Hospital following the World Health Organization criteria (HbA1c and fasting plasma glucose levels) and were drawn from the department’s clinical population. Inclusion criteria were: (1) between 40–75 years of age, (2) T2DM for at least 1 year prior, and (3) informed consent. Diagnosis and severity of diabetic retinopathy were determined by hospital experts based on a technical report according to the Early Treatment Diabetic Retinopathy Study guidelines.

Control subjects were recruited from the general population of the Hospital or University staff. Inclusion criteria were: (1) between 40–75 years of age, (2) T2DM diagnosis was ruled out (based on HbA1c and fasting plasma glucose levels), and (3) informed consent.

Eye and hand dominance were assessed using the hole-in-the-card test (Dolman method) and the Edinburgh inventory, respectively. All participants had either normal or corrected-to-normal vision and no history of any neurological or psychiatric disease. Fundus photography and optical coherence tomography were used to confirm the visual system’s health.

A neuroradiologist assessed standard clinical 2D-FLAIR and 3D-SPACE T2-weighted scans for the presence of white matter hyper-intensities. Participants with vascular or structural lesions or any confounding changes were excluded. Participants with a maximum head movement of more than 3 mm during acquisition, as detected by motion-correction, were also excluded.

After exclusion, 51 T2DM and 67 control subjects were selected. Participants were randomly divided into two datasets: Dataset 1 (DS1), containing 80% of all participants, and Dataset 2 (DS2), containing the remaining 20%. Two constraints were imposed on the split: (1) the same proportion of T2DM subjects was forced in both sets; (2) for DS2, the age and gender distributions between T2DM and control subjects were forcibly matched, i.e., we randomly selected either a T2DM or control subject and then found the closest match in terms of gender and age from the opposite class.

### 2.3 Experimental design and imaging

All participants were imaged at the facilities of the Portuguese Brain Imaging Network in a 3 Tesla scanner (Magnetom TIM Trio, phased array 12-channel birdcage head coil; Siemens, Munich, Germany). Imaging comprised magnetic resonance (MR) anatomical and functional scans. Three-dimensional anatomical MPRAGE (magnetization-prepared rapid gradient echo) scans were acquired using a standard T1-weighted gradient echo (GE) pulse sequence (2,530 *ms* repetition time (TR); 3.42 *ms* echo time (TE); 1,100 *ms* inversion time (TI); 7° flip angle; 176 slices; 1 × 1 × 1 *mm* voxel size; 256 *mm* field of view (FOV)).

The functional series consisted of a run of 116 GE, EPI scans (2,500 *ms* TR; 30 *ms* TE; 90° flip angle; 36 interleaved slices, 3 × 3 × 3 *mm* voxel size; 256 *mm* FOV) in an event-related design stimulation paradigm with measurement of the BOLD signal.

#### 2.3.1 Event-related task: stimuli description

Subjects were asked to compare the speed of two white dots, a reference, and a target dot, with 22.9 *cd*/*m*^2^ mean luminance moving on a gray background (9.39 *cd*/*m*^2^ mean luminance) and to select the fastest of the two.

Stimuli setup was prepared in MATLAB using the Psychophysics Toolbox (David Brainard and Denis Pelli, http://www.psychtoolbox.org) and projected with an LCD projector (Avotec Real Eye Silent Vision 6,011, 1,024 × 768 pixels, 60 *Hz* refresh-rate; Avotec Incorporated, Stuart, FL, USA) onto a screen pad positioned in the bore at 163 *cm* (22.62° × 17.06° image size; mirror 50 *cm* from the screen). Stimuli were viewed with one eye while the other eye was covered with an opaque patch.

Two dots (0.22° × 0.22° dot size, 0.33° × 0.33° fixation-cross size) were presented, one in each visual hemifield, randomly distributed at 7.5° eccentricity along the horizontal meridian, moving back and forth along 2° path in a pseudo-random linear trajectory (between 0 and 180°). The subject selects the faster moving dot by pressing a button on a Cedrus Lumina LP-400, LU400 PAIR response box (Cedrus Corporation, San Pedro, CA, USA).

#### 2.3.2 Speed threshold discrimination task

Prior to functional acquisition, each subject’s speed discrimination threshold was determined, i.e., the limit delta between a reference and target dot at which each participant was able to correctly discriminate the faster dot. This was done inside the scanner with the dominant eye. The dots were in view for 400 ms. Successive trials were performed with decreasing delta. The speed of the target dot on successive trials was determined using a descending logarithmic staircase. The staircase had six reversals (two practice/four experimental). It allowed us to compute speeds from 24°/*s* (initial target speed) to 5°/*s* (reference speed) using a step value ranging from 1–0.05 dB. The threshold was estimated using the average of the last four reversals.

#### 2.3.3 Stimulation conditions

Functional acquisition for the event-related design stimulation paradigm included two conditions, designated threshold and sub-maximum, each presented 10 times per hemifield to increase statistical power. For the threshold condition the target dot moved at the reference speed plus the discrimination threshold (individually determined); and for the sub-maximum condition the target dot moved at the reference speed plus three times the same threshold. Note that in both conditions the reference dot moved at the reference speed (5°/*s*).

Each stimulation period lasted 400 ms, and the baseline fixation period was randomly assigned to be either 4,600, 7,100, or 9,600 ms. Participants were instructed to maintain fixation on the fixation-cross. The stimulation and imaging protocol have been further described in Duarte et al. (2015).

### 2.4 Data analysis

#### 2.4.1 Pre-processing

A standard sequential fMRI processing protocol was carried out using FSL (FMRIB Software Library v6.0, Analysis Group, FMRIB, Oxford, United Kingdom) which included slice scan time correction, temporal high-pass filtering, inter-scan head motion-correction, slight spatial-smoothing, and normalization to standard MNI152 space. Normalization to the standard MNI152 space enables inter-patient comparisons and was used to analyze and interpret the results obtained.

Scans were cropped around each stimulation period starting from the scan immediately before stimulation and covering the stimulation plus 10 s ([−2.5:10*s*]). Scans were averaged for each condition and hemifield, resulting in 4D matrices of 90 × 90 × 90 voxels ×8 TRs. All data were normalized to have zero mean and unit variance. Each condition and hemifield was trained and tested separately.

#### 2.4.2 Data availability statement

High-quality medical data are difficult to produce, especially when dealing with expensive and time-consuming data sources such as fMRI. Although our study population was larger than similar literature, dataset size is still key when applying DL. As overfitting mitigation tools, we chose a slim network architecture, limiting trainable parameters, used dropout regularization to simulate an ensemble of NNs with similar yet distinct model configurations, and incorporated artificial data augmentation per batch to increase training variability.

As the name implies, data augmentation is an approach to artificially expand the dataset by generating new samples from existing ones to introduce variability through various manipulations. In this work, we used both standard and specially designed operations. Random translations, rotations, and normal noise addition were used. In addition, we randomly sampled repetitions for averaging, i.e., instead of averaging all 10 repetitions per hemifield per condition, a random combination of between 7 and 9 repetitions was averaged instead (randomly selected from 175 total combinations).

#### 2.4.3 Classification models

CNNs are the gold-standard for image classification (Alzubaidi et al., 2021). This type of network consists of several layers, but is characterized by, and therefore named after, the convolutional layer. This layer uses scanning kernels to convolve the input, which increases its efficiency.

A deep CNN can create more complex abstractions and theoretically solve more complex classification tasks. However, the problem of exploding/vanishing gradients arises. The ResNet architecture, proposed in He et al. (2016), introduced shortcut connections, which mitigate these problems. These shortcut connections are added to the outputs of the stacked layers. ResNet is a modular architecture that stacks multiple residual blocks, i.e., layer groups composed of stacked layers and a shortcut connection that skips them.

The architecture used consists of a 3D convolutional layer followed by four residual blocks and is schematically shown in the Supplementary materials. Batch normalization (BN) and ReLu (Rectified Linear Unit) activation are used to improve stability and introduce non-linearity in the training process, respectively. All convolutions use a stride of one and 3 × 3 kernels. Within the residual blocks there is a max-pooling layer to halve the feature maps using a 2 × 2 kernel and stride two. The final classification block includes a fully connected layer, dropout, and a sigmoid activation function to classify each scan.

#### 2.4.4 Training and testing

All scans belonging to DS2 subjects were used for testing only. This separation ensures the independence of testing and the validity of the results. For each model, 10% of DS1 scans were used for hyperparameter tuning. The binary cross-entropy cost-function was used as the loss, which was backpropagated to update the weights of the NN using the stochastic gradient descent optimizer algorithm.

**Figure 1:**
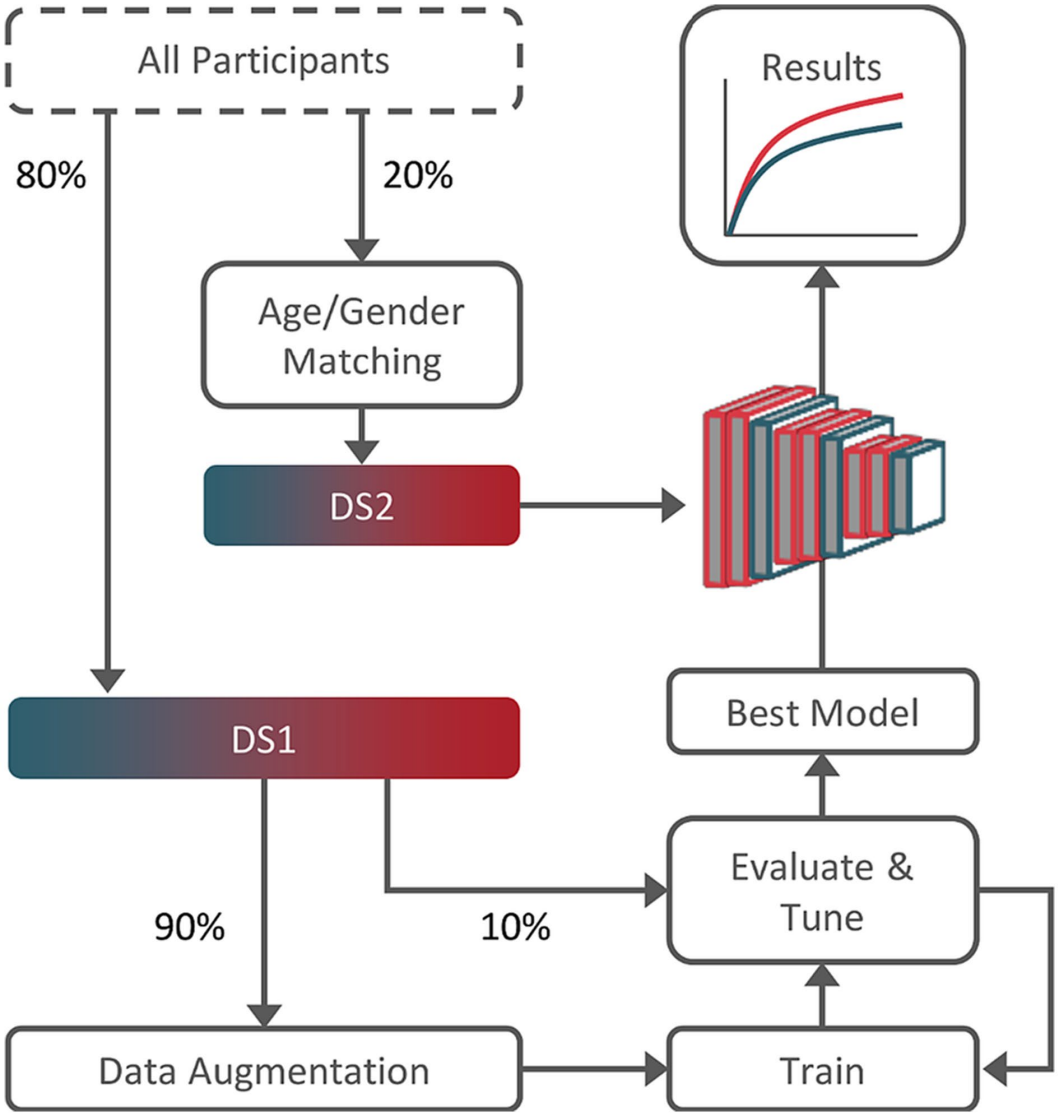
Training, tuning, and testing workflow. Dataset 2 (DS2) subjects were age and gender-matched guarantying results validity. Dataset 1 (DS1), composed of the remainder 80% of the subjects, was used for training and tuning (90%/10% split).

Results on DS2 were obtained using the best performing hyperparameter combination, evaluated independently for each model in the respective tuning set: grid-search to select dropout-rate, learning-rate, momentum, and training steps. Figure 1 summarizes the study workflow.

All models were built, trained, and tested in Python 3.7.9 using the Keras framework with TensorFlow backend. Weights were initially set using a Glorot uniform initializer and biases were set to zero. Network training was performed on a desktop equipped with an AMD Ryzen 93,900x CPU @3.8 GHz, 64 GB RAM and a Nvidia RTX 3080, using CUDA version 11.5.

#### 2.4.5 Statistical evaluation

To evaluate testing, performance metrics were chosen for the problem at hand and the distribution of the labels in the dataset. The area under the receiver operating characteristic curve (AUC), accuracy, and F1-score were computed. In binary classification tasks these are commonly used to evaluate the performance of a model. Balanced accuracy, sensitivity, and specificity have also been computed.

#### 2.4.6 Interpretability

One of the main drawbacks of non-linear methods like DL is their “black-box” nature. Despite the superior performance shown on many discrete problems, the lack of transparency limits its explainability and hinders its use. Explainable DL is a milestone for clinical translation.

There are several approaches to improve the interpretability of DL methods. Here, we apply deep Taylor decomposition (DTD) (Montavon et al., 2017). The rationale is to decompose the final decision into the individual contributions of each given input by means of relevance backpropagation, i.e., to trace-back contributions from output to input by Taylor decomposition. Ultimately, heatmaps are generated for each classification decision, representing the relative relevance of each voxel to the final classification. Leveraging the individual transformation matrices to the standardized MNI152 space, we transform and average the heatmaps to derive a global response. This approach allows us to identify the brain structures/regions that are more important for classification.

#### 2.4.7 Cluster-based analysis

The heatmaps can be further processed to define clusters. We applied an empirically determined threshold to the average heatmap, and defined clusters based on neighborhood connectivity. These were further processed with morphological erosion followed by dilation (morphological open operation). Clusters smaller than 20 voxels were ignored. The resulting relevance clusters can be used as regions of interest (ROIs) for subsequent analysis.

We applied deconvolution analysis within the defined ROIs to separate the contributions of the different events. Instead of one predictor per condition, a fixed number of shifted stick predictors are defined, covering the 20 s post-stimulus. It is an alternative to traditional GLM analysis. Its main advantage is its flexibility, since the HRF is not defined *a priori*, but is instead estimated for each event type. This is relevant in the case of diabetes, where standard HRF is not guaranteed.

## 3 Results

### 3.1 Data characteristics

A total of 51 T2DM and 67 control subjects were selected. Participants were randomly divided into two datasets: DS1 used for training and tuning of the network, and DS2 used for testing. DS2 is age- and gender-matched, with a mean age of 50.6 ± 1.4 and 51.6 ± 1.8 years for controls and T2DM subjects, respectively. The complete characterization of DS1 and DS2 is available in the Supplementary materials. All participants underwent three-dimensional anatomical MPRAGE scanning and event-related task-based functional scanning. For the latter, patients were asked to compare the speed of two white dots, a reference and a target dot, and to select the fastest of the two. Overall response accuracy during scanning did not show a significant difference between controls and T2DM subjects (data not shown). This study was conducted under performance-matched conditions with physically matched stimuli.

### 3.2 fMRI data reveal changes in the brain of patients

**Figure 2:**
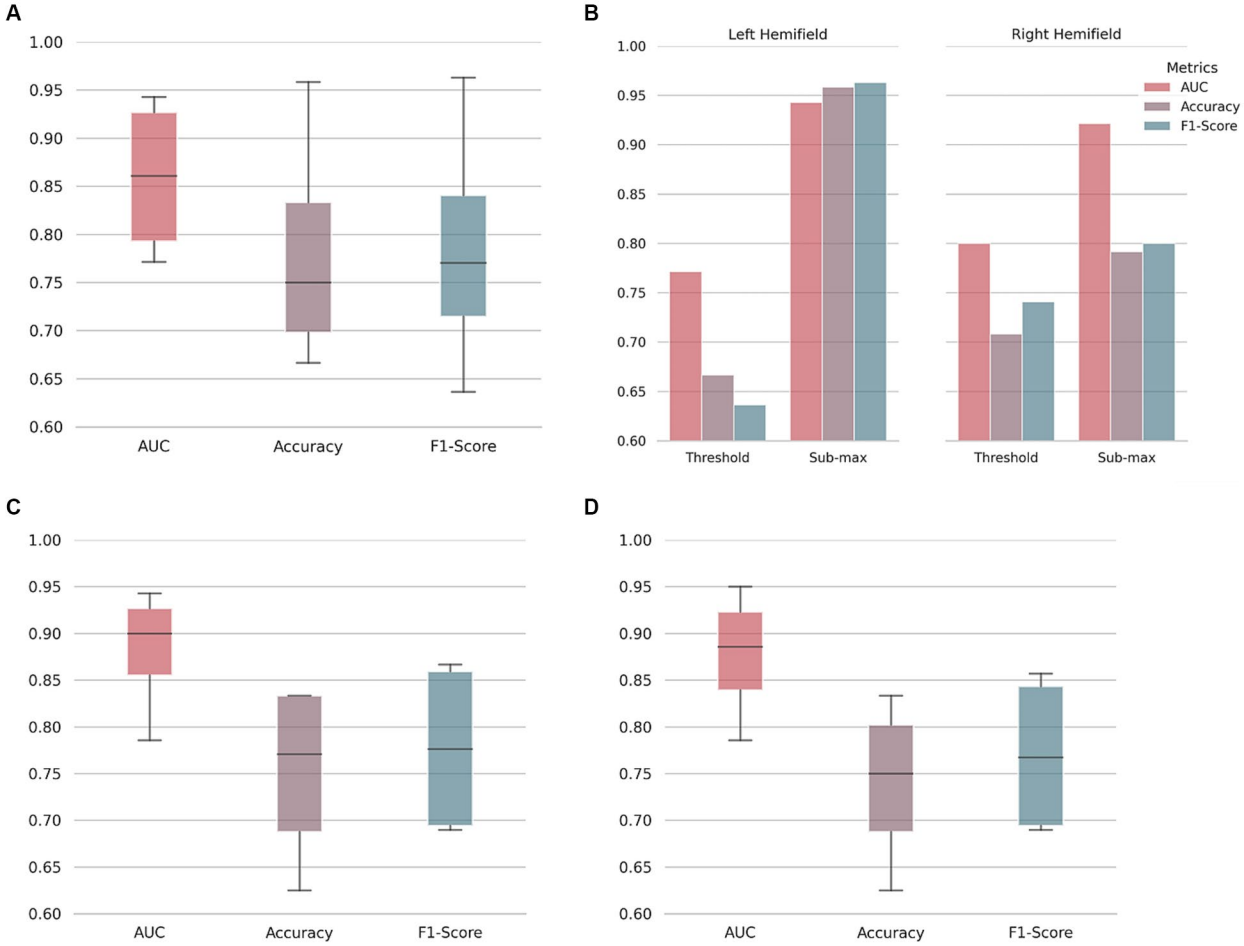
Type 2 diabetes mellitus classification performance. On **(A)**, area under the receiver operating characteristic curve (AUC), accuracy, and F1-score boxplots. Median, first and third quartile represented. Whiskers at each quartile plus 1.5 times the inter quartile range. On **(B)**, discriminated values per hemifield and stimulus condition (at psychophysical threshold level, or submaximal motion contrast level) as indicated. Note the better performance for the left hemifield, corresponding to the right hemisphere. To understand the influence of pre-processing and/or in-scanner head movement, a neural network was trained on raw/unprocessed data and then used to classify both unprocessed **(C)** and motion-corrected data **(D)**.

Motion-corrected DS1 scans were used to train and tune a CNN to discriminate between T2DM and control scans. The performance was evaluated on DS2 motion-corrected scans. The AUC, accuracy, and F1-score results per condition and hemifield are shown in Figures 2A,B.

As shown, performance ranges from a minimum of 0.771 AUC and 66.7% accuracy (threshold motion contrast condition, right hemifield) to 0.943 AUC and 95.8% accuracy (sub-maximum motion contrast condition, left hemifield). Discrimination performance for the sub-maximum condition is better than for the threshold condition in both hemifields, as expected from the larger stimulus signal to noise ratio (serving also as a sanity check). Our results indicate that there are measurable changes in the fMRI signals of T2DM patients compared to controls. Balanced accuracy, sensitivity, and specificity results are shown in the Supplementary materials.

### 3.3 Head movement does not affect performance

DL is a broad-spectrum classifier. With NNs, layer by layer increasingly more complex representations are created, and, theoretically, no feature representation is off-limits. Despite the exclusion criteria used (subjects with in-scanner head movement greater than 3 *mm* were excluded), it is important to ensure that neither the head movement in the scanner, nor the pre-processing typically applied to correct for it, is partially responsible for the discrimination performance.

To understand the influence of pre-processing and/or in-scanner head movement, data were compared at two processing stages: unprocessed (or raw) and motion-corrected. A CNN was trained and tuned with raw fMRI data. This network was then used to classify both raw and motion-corrected data (Figures 2C,D). As shown, although there is some variability, similar test performance was achieved for the two processing stages. Results suggest that our classification approach has no specific bias due to pre-processing or in-scanner head movement up to the tested limit of 3 *mm*.

### 3.4 Backpropagation shows relevant brain regions

**Figure 3:**
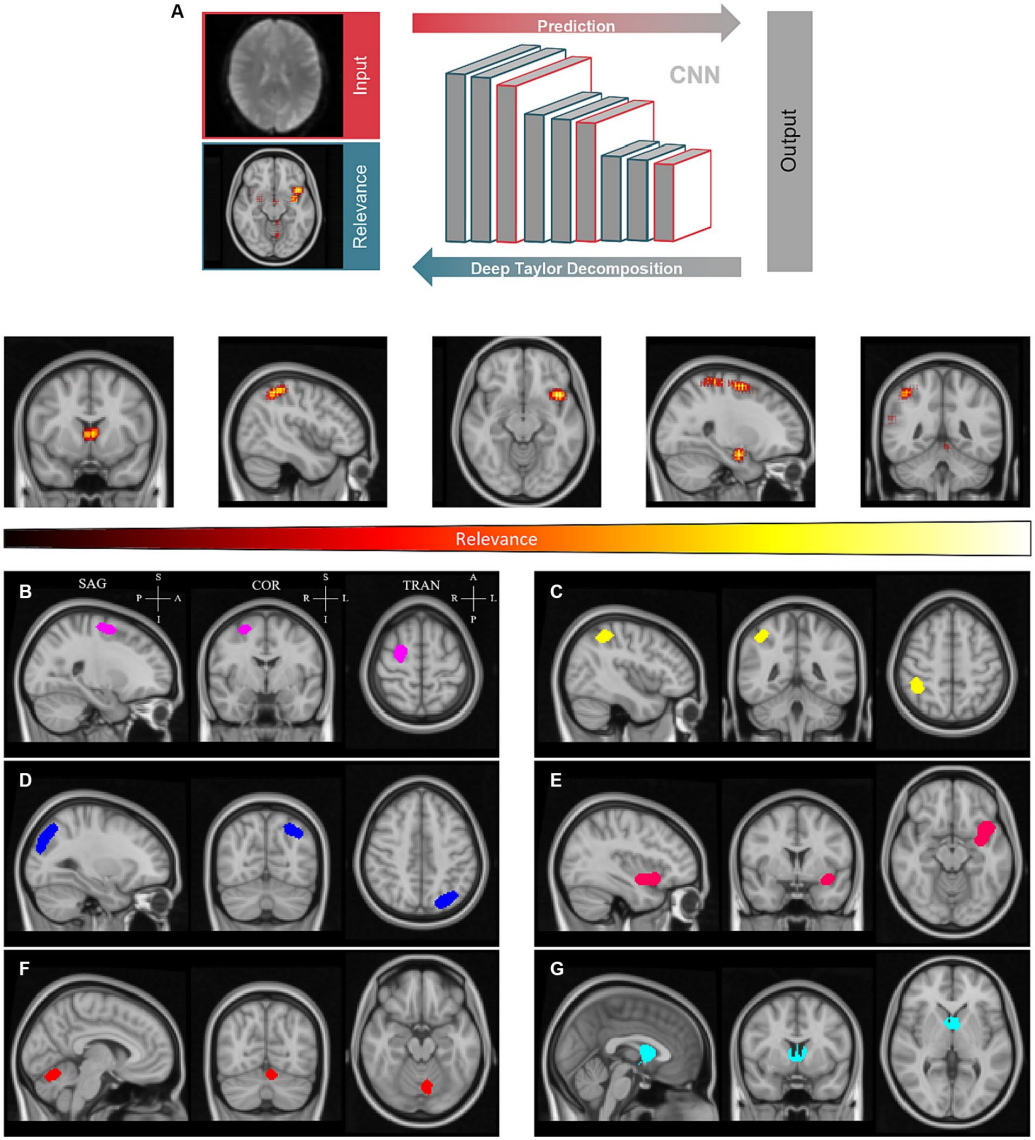
Clustering of relevance heatmaps computed by deep Taylor decomposition to define regions of interest. **(A)** - Individual pixel contributions were backpropagated over the convolutional neural network (CNN) to generate relevance heatmaps. **(B-G)** - Averaged heatmaps were thresholded and labeled to create relevance clusters. Sagittal (SAG), coronal (COR), and transverse (TRA) planes are shown for each cluster. These are located approximately in the superior frontal gyrus **(B)**, angular gyrus **(C)**, extrastriate visual and parietal cortex **(D)**, insula **(E)**, cerebellum **(F)**, and thalamus **(G)**, left to right and, top to bottom, respectively. Clusters are represented with distinct colors for the sake of convenience. P – Posterior; A – Anterior; S -Superior; I – Inferior.

To shed light on the decision process, we derived the individual pixel contributions using DTD (Montavon et al., 2017) and generated relevance heatmaps (Figure 3).

The resulting heatmaps were then transformed into standardized MNI152 space to be averaged across subjects. Average relevance patterns calculated independently for controls and T2DM subjects showed similar patterns (see Supplementary materials). From the resulting average heatmaps, we could discern six semi-consistent agglomerations across the four models (two conditions per hemifield). A global average relevance heatmap was computed and clusters were determined (Figures 3B–G). As shown, clusters are located approximately in the superior frontal gyrus, angular gyrus, visual cortex, insula, cerebellum, and thalamus. The sagittal, coronal, and transverse planes are shown for each cluster, centered on the peak relevance voxel. The location of the peak relevance voxel (MNI152 space) and volume for each cluster are shown in the Supplementary materials.

### 3.5 Cluster-based hemodynamic response function

**Figure 4:**
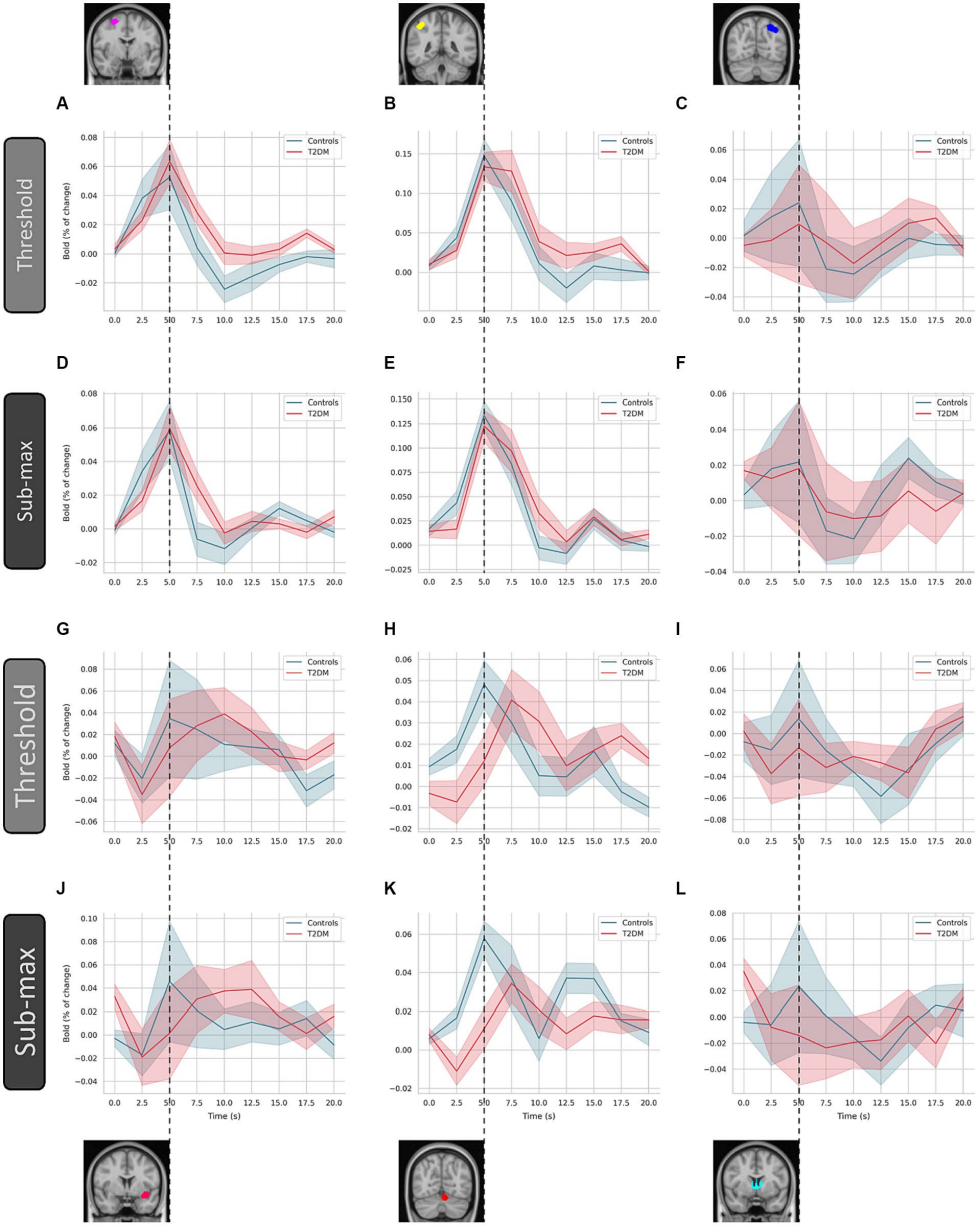
Estimated hemodynamic response function by deconvolution-based analysis. The hemodynamic response function was computed over the six indicated clusters for controls and type 2 diabetes patients (T2DM). These are (left to right, top to bottom) the superior frontal gyrus (**A** and **D**), angular gyrus (**B** and **E**), visual cortex (**C** and **F**), insula (**G** and **J**), cerebellum (**H** and **K**), and thalamus (**I** and **L**), respectively. Average and standard deviation are represented (line and shaded area, respectively), for each condition (threshold and sub-maximum as indicated). Dashed line highlights the time of peak response of control subjects.

We resorted to deconvolution-based GLM analysis to more accurately represent the time course of the neural activity estimated from the measured BOLD response. In Figure 4, we show the global average of the estimated HRF for each group (controls and T2DM subjects), computed for both threshold and sub-maximum events (in terms of speed contrast) and over the six derived clusters. In the specific case of the deconvolution GLM, the beta weights of the GLM allow the reconstruction of the entire BOLD response.

In T2DM patients, the characteristic undershoot of the HRF before returning to baseline seems to be less pronounced compared to controls (Figures 4A–D). The HRF peaks lower in T2DM subjects in most ROIs. However, the ROI located in the superior frontal gyrus appears to be an exception (Figures 4A–D). Although the general shape is similar between controls and TD2M subjects, the HRF of the latter appears to be delayed compared to controls. This effect, present in several ROIs, is most pronounced in Figures 4G,H.

## 4 Discussion

For the first time, we have combined event-related task-based fMRI imaging, deconvolution GLM analysis, and novel DL methods. This innovative methodology offers a unique perspective to validate in a data-driven manner changes in the BOLD response of T2DM patients. Neither DL nor deconvolution GLM use any *a priori* information. With this fully data-driven approach, we unbiasedly demonstrated that T2DM patients have an altered hemodynamic event-related response compared to age- and gender-matched controls, which may therefore represent a potential disease biomarker.

We used DL to model normal and T2DM task-based (visual speed discrimination) fMRI scans. The obtained results show that discrimination between the two groups was possible and accurate. The model trained on the sub-maximum condition (the condition with the highest speed contrast) presented on the left hemifield accurately predicted over 95% of the scans. This is reassuring since optimal classification is attained using the best stimulus contrast, and at the hemisphere that dominates for spatial vision, thereby confirming biological plausibility. For the threshold condition (lowest speed contrast, left hemifield), the trained model had an accuracy rate of 66.7%. These hemifield differences are physiologically meaningful and might be explained by known hemispheric differences in the cortical spatial processing (Silva et al., 2008). The left visual hemifield corresponds to the brain’s right hemifield, which dominates in cortical visuospatial processing (Silva et al., 2008). Performance variability may also reflect task complexity.

To investigate the influence of both the head motion in the scanner and/or the preprocessing applied to correct it, a CNN was trained and tuned on raw/unprocessed fMRI data and then used to classify both unprocessed and motion-corrected data. The performance of the models was similar in both cases. The performance of the network trained on raw data and the network trained on motion-corrected data was also comparable. These results suggest that the influence is negligible.

The relevance clusters obtained from the DTD heatmaps were used as ROIs for subsequent deconvolution analysis to understand the differences between T2DM patients and control subjects. T2DM patients showed an altered hemodynamic response. The results of this data-driven approach are consistent with our hypothesis proposed in Duarte et al. (2015, 2023). In response to increased neuronal activation, there is an increase in oxygen demand and, consequently, an increase in blood supply. In diseased subjects, this autoregulatory system may be disrupted, resulting in alterations in the standard BOLD signal and HRF. Physiological changes in the brain vasculature, prior to any evidence of over structural lesions, may affect the normal response. The demonstrated HRF differences challenges the conventional analysis of task-based fMRI in T2DM patients with the standard HRF and its appropriateness in neurocognitive paradigms. These altered HRF patterns are promising biomarkers that may lead to a better understanding of the neurophysiological changes in T2DM. Overall, we demonstrated the potential of DL to provide novel insight into neuropathology and highlighted the need for interpretable DL methods in healthcare research.

### 4.1 Strengths and limitations

MR structural and functional imaging are indispensable tools in the research, diagnosis, and follow-up of neuropathology. However, MR remains a time- and labor-intensive data source which inherently limits data availability. Task-based protocols increase specificity but intrinsically restrict inter-study sharing. One of the reasons why the largest datasets rely on resting state. However, resting state cannot directly capture stimulus-related functional properties and event-related responses. These are critical for unraveling HRFs. Given these limitations, our study had a larger sample size than similar studies. However, it is important to note that a more extensive sample size would further strengthen the study’s findings. This study serves as an initial step, highlighting the need for future research and to encompass larger cohorts. Mainly, the integration of multicentric datasets covering the entire spectrum of T2DM will facilitate the development of more robust methodologies and conclusions.

Because NNs are powerful but indiscriminate learners, controlling bias is crucial. We aimed to maximize the participant pool, while ensuring the validity of the results. DS2, for testing, was age and gender-matched, while DS1, for training and tuning, was not, which is less critical, but may impact performance. The performance achieved is valid but could ultimately be improved further.

Despite the performance advantage of DL against other machine learning methods, the lack of transparency inherent to non-linear methods means that one cannot know what factors its decision is being made upon. The biological plausibility of classification performance is addressed above. In this work, we applied DTD to derive heatmaps representing the relative importance of each voxel for the classification between controls and T2DM. Functional scans were co-registered with structural MRI scans and, in turn, normalized to the MNI152 space. This allowed us to transform the resulting heatmaps to standardized space for group analysis. Inter-subject averaging revealed relevance clusters located in the superior frontal and angular gyrus (Zhou et al., 2010; García-Casares et al., 2014; Tan et al., 2019; Qi et al., 2021), visual extrastriate cortex (Newsome and Pare, 1988; Yao et al., 2021), insula (Chang et al., 2006; Xia et al., 2017), cerebellum (Chen et al., 2015; Yao et al., 2021), and thalamus (Chen et al., 2015), all regions related to the disease and/or task (speed discrimination processing and perceptual decision). It is revealing that these regions were of more importance in this data-driven approach, as these are consistent with previous observations in T2DM, which helps to substantiate the classification obtained.

## Data availability statement

The raw data supporting the conclusions of this article will be made available by the authors upon formal and reasonable request.

## Ethics statement

The studies involving humans were approved by Ethics Committee of the Faculty of Medicine of the University of Coimbra. The studies were conducted in accordance with the local legislation and institutional requirements. The participants provided their written informed consent to participate in this study.

## Author contributions

PG: Conceptualization, Data curation, Formal analysis, Investigation, Methodology, Software, Validation, Visualization, Writing – original draft. PS: Formal analysis, Methodology, Visualization, Writing – review & editing. JD: Data curation, Formal analysis, Writing – review & editing. JC: Data curation, Formal analysis, Writing – review & editing. CM: Data curation, Resources, Writing – review & editing. LG: Data curation, Resources, Writing – review & editing. RB: Formal analysis, Methodology, Supervision, Writing – review & editing. MC-B: Conceptualization, Formal analysis, Funding acquisition, Methodology, Project administration, Resources, Supervision, Validation, Writing – review & editing.
