# Supplementary material for: The hemodynamic response function as a type 2 diabetes biomarker: a data-driven approach
Source: Front Neuroinform. 2024 Jan 5;17:1321178. doi: 10.3389/fninf.2023.1321178 (PMC10796780; doi:10.3389/fninf.2023.1321178)
Supplement: Supplementary file 1 [file Data_Sheet_1.docx]

Supplementary Material

The hemodynamic response function as a type 2 diabetes biomarker: a data-driven approach

**Pedro Guimarães,^1, *^ Pedro Serranho,^1,2^ João Valente Duarte,^1,3^ Joana Crisóstomo,^1^ Carolina Moreno,^4^ Leonor Gomes,^4^ Rui Bernardes,^1,5^ and Miguel Castelo-Branco^1,5^**

^1^University of Coimbra, Coimbra Institute for Biomedical Imaging and Translational Research (CIBIT), Institute for Nuclear Sciences Applied to Health (ICNAS), Coimbra, Portugal

^2^Universidade Aberta, Department of Sciences and Technology, Lisboa, Portugal

^3^University of Coimbra, Faculty of Medicine (FMUC), Coimbra, Portugal

^4^Department of Endocrinology, University Hospital of Coimbra (CHUC), Coimbra, Portugal

^5^University of Coimbra, Clinical Academic Center of Coimbra (CACC), Faculty of Medicine (FMUC), Coimbra, Portugal

*pedro.guimaraes@icnas.uc.pt


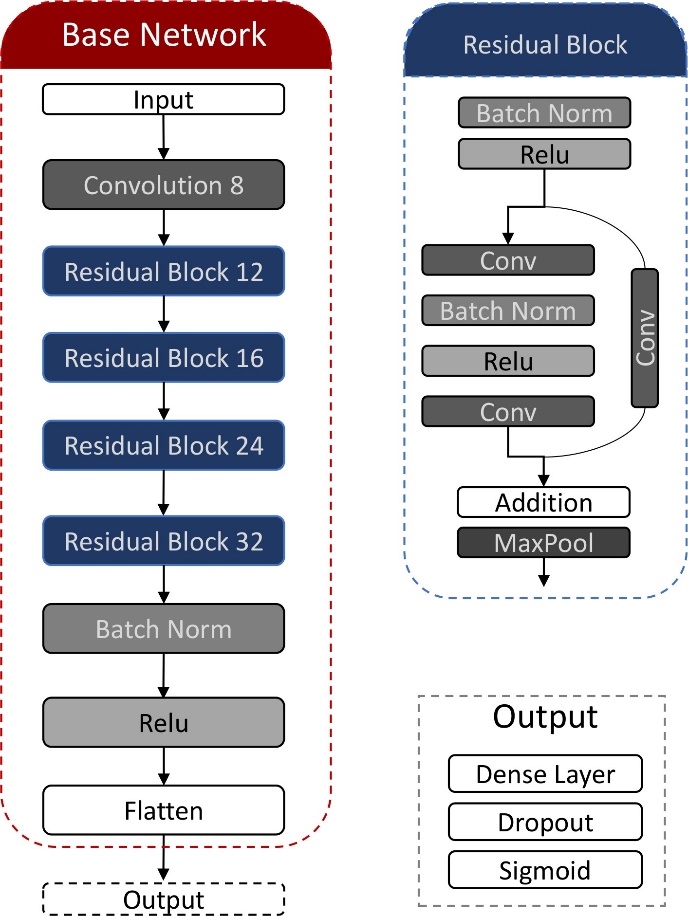


Supplementary Figure 1. Schematic representation of the proposed 3D convolutional neural network. The network is based on the ResNet architecture and composed of 4 residual blocks, also illustrated (blue-dashed box). The output block (gray-dashed box) by a fully connected layer, the dropout layer, and a sigmoid activation function.


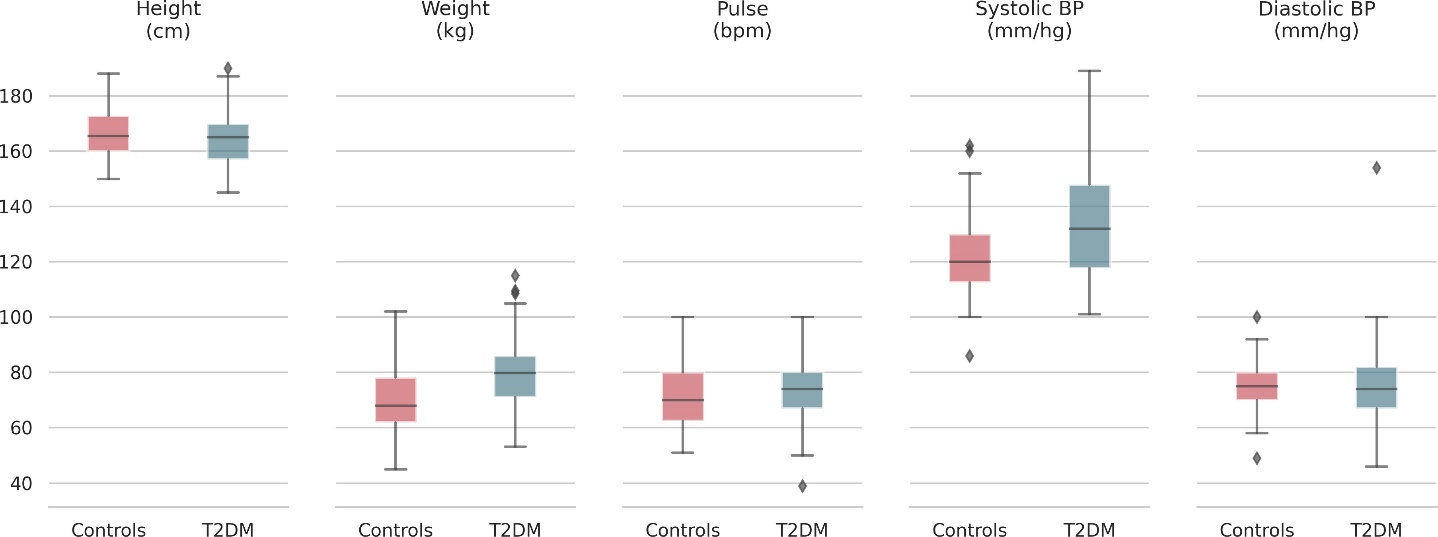


Supplementary Figure 2. Data characteristics. Height, weight, pulse, and systolic and diastolic blood pressure boxplots, respectively, for controls and type 2 diabetes mellitus (T2DM) subjects. Median, first and third quartile represented. Whiskers at each quartile plus 1.5 times the inter quartile range. Outliers showed as diamonds.


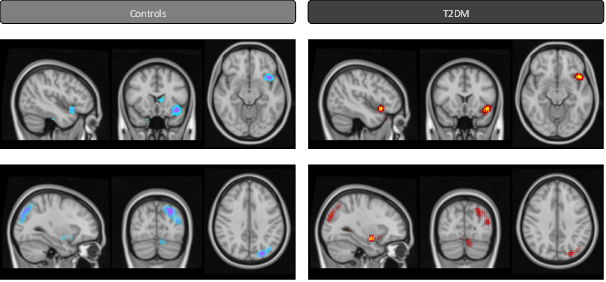


**Supplementary Figure 3.** Average relevance patterns. Representative average relevance patterns calculated independently for control and type 2 diabetes mellitus (T2DM) subjects. Individual pixel contributions were backpropagated through the convolutional neural network to generate heatmaps. The pseudocolor for control and TD2M subjects is different to facilitate visualization. As shown, both groups show similar patterns of relevance.

**Supplementary Table 1.** Additional performance metrics. Balanced accuracy, sensitivity, and specificity discriminated per hemifield and stimulus condition (at psychophysical threshold level, or submaximal motion contrast level) as indicated.

|  | **Left Hemifield** | | **Right Hemifield** | |
| --- | --- | --- | --- | --- |
|  | **Threshold** | **Sub-max** | **Threshold** | **Sub-max** |
| Balanced Accuracy | 0.70 | 0.96 | 0.71 | 0.81 |
| Specificity | 0.50 | 0.93 | 0.71 | 0.71 |
| Sensitivity | 0.90 | 1.00 | 0.70 | 0.90 |

**Supplementary Table 1.** Dataset characterization. Dataset 1 (DS1) and 2 (DS2) characterization by group, controls and type 2 diabetes mellitus (T2DM) subjects.

|  | **Dataset 1 (DS1)** | | **Dataset 2 (DS2)** | |
| --- | --- | --- | --- | --- |
|  | **Controls** | **T2DM** | **Controls** | **T2DM** |
| Count | 53 | 39 | 14 | 12 |
| Age (Years) | 48.528 (8.065) [40, 71] | 61.415 (7.071) [45, 73] | 50.643 (1.445) [49, 54] | 51.600 (1.800) [49, 54] |
| T2DM Duration (Years) | - | 14.658 (7.915) [1, 39] | - | 16.625 (9.068) [4, 31] |
| Gender | 41.5 / 58.5 % (Female / Male) | 56.1 / 43.9 % (Female / Male) | 42.9 / 57.1 % (Female / Male) | 41.7 / 58.3 % (Female / Male) |
| Eye Dominance | 41.5 / 58.5 % (Left / Right) | 34.1 / 65.9 % (Left / Right) | 42.9 / 57.1 % (Left / Right) | 40.0 / 60.0 % (Left / Right) |
| Writing Hand | 3.8 / 96.2 % (Left / Right) | 0.0 / 100.0 % (Left / Right) | 7.1 / 92.9 % (Left / Right) | 0.0 / 100.0 % (Left / Right) |
| Height (m) | 1.661 (0.093) [1.530, 1.880] | 1.625 (0.094) [1.450, 1.850] | 1.671 (0.077) [1.500, 1.800] | 1.638 (0.148) [1.450, 1.900] |
| Weight (kg) | 69.835 (13.983) [45, 102.000] | 78.539 (12.084) [53.100, 104.000] | 71.236 (9.328) [62.000, 90.000] | 80.320 (18.302) [53.200, 115.000] |
| BMI | 25.102 (3.360) [18.500, 33.400] | 29.839 (4.890) [22.100, 43.700] | 25.486 (2.554) [21.200, 29.100] | 29.850 (5.039) [20.500, 34.900] |
| Abd. Perimeter (cm) | 87.000 (9.557) [74.000, 104.000] | 100.812 (14.231) [71.000, 140.000] | 84.625 (10.206) [74.000, 96.500] | 99.375 (9.974) [81.000, 110.000] |
| Pulse (bpm) | 70.071 (13.724) [51, 100] | 72.439 (10.693) [39, 90] | 73.750 (15.990) [53, 93] | 84.222 (9.986) [70, 100] |
| Systolic BP (mmHg) | 118.316 (10.945) [100.000, 146.000] | 135.732 (20.357) [101.000, 189.000] | 119.200 (6.735) [109.000, 130.000] | 135.556 (14.423) [114.000, 158.000] |
| Diastolic BP (mmHg) | 73.579 (5.807) [60.000, 80.000] | 74.317 (12.569) [46.000, 100.000] | 75.000 (8.319) [61.000, 86.000] | 83.667 (8.472) [68.000, 94.000] |
